# Improving geographical accessibility modeling for operational use by local health actors

**DOI:** 10.1186/s12942-020-00220-6

**Published:** 2020-07-06

**Authors:** Felana Angella Ihantamalala, Vincent Herbreteau, Christophe Révillion, Mauricianot Randriamihaja, Jérémy Commins, Tanjona Andréambeloson, Feno H. Rafenoarimalala, Andriamihaja Randrianambinina, Laura F. Cordier, Matthew H. Bonds, Andres Garchitorena

**Affiliations:** 1 NGO PIVOT, Ranomafana, Madagascar; 2 Department of Global Health and Social Medicine, Harvard Medical School, Boston, USA; 3 Institut de Recherche pour le Développement, UMR 228 Espace-Dev (IRD, UA, UG, UM, UR), Phnom Penh, Cambodia; 4 Université de La Réunion, UMR 228 Espace-Dev (IRD, UA, UG, UM, UR), Saint-Pierre, La Réunion France; 5 School of Management and Technological Innovation, University of Fianarantsoa, Fianarantsoa, Madagascar; 6 Ministry of Health in Madagascar, Antananarivo, Madagascar; 7 MIVEGEC, Univ. Montpellier, CNRS, IRD, Montpellier, France

**Keywords:** Madagascar, Universal health coverage, Geographic barriers, e-Health tools

## Abstract

**Background:**

Geographical accessibility to health facilities remains one of the main barriers to access care in rural areas of the developing world. Although methods and tools exist to model geographic accessibility, the lack of basic geographic information prevents their widespread use at the local level for targeted program implementation. The aim of this study was to develop very precise, context-specific estimates of geographic accessibility to care in a rural district of Madagascar to help with the design and implementation of interventions that improve access for remote populations.

**Methods:**

We used a participatory approach to map all the paths, residential areas, buildings and rice fields on OpenStreetMap (OSM). We estimated shortest routes from every household in the District to the nearest primary health care center (PHC) and community health site (CHS) with the Open Source Routing Machine (OSMR) tool. Then, we used remote sensing methods to obtain a high resolution land cover map, a digital elevation model and rainfall data to model travel speed. Travel speed models were calibrated with field data obtained by GPS tracking in a sample of 168 walking routes. Model results were used to predict travel time to seek care at PHCs and CHSs for all the shortest routes estimated earlier. Finally, we integrated geographical accessibility results into an e-health platform developed with R Shiny.

**Results:**

We mapped over 100,000 buildings, 23,000 km of footpaths, and 4925 residential areas throughout Ifanadiana district; these data are freely available on OSM. We found that over three quarters of the population lived more than one hour away from a PHC, and 10–15% lived more than 1 h away from a CHS. Moreover, we identified areas in the North and East of the district where the nearest PHC was further than 5 h away, and vulnerable populations across the district with poor geographical access (> 1 h) to both PHCs and CHSs.

**Conclusion:**

Our study demonstrates how to improve geographical accessibility modeling so that results can be context-specific and operationally actionable by local health actors. The importance of such approaches is paramount for achieving universal health coverage (UHC) in rural areas throughout the world.

## Background

In 2018, world leaders celebrated the 40 years since the adoption of the Alma Ata Declaration, which recognized the need to invest in primary care as the key to attaining the goal of “Health for All”. While we have witnessed since then an unprecedented global improvement in health indicators, half of the world’s population continues to lack access to essential health services [1]. Low health care access in rural settings of the developing world is due to a combination of financial, geographical and health system barriers [2–6]. To reduce the impact that weak health systems and user-fees have on health care access, there is a growing consensus around the central importance of sector-wide approaches such as health system strengthening (HSS) and universal health coverage (UHC) [7–9]. An increasingly recognized pillar of many HSS and UHC efforts is the role of community health workers (CHWs) to reduce geographical inequities in access and ensure the delivery of primary care at the community level [10].

Community health strategies are underway in most rural areas of Sub-Saharan Africa, where health infrastructure is sparse and the majority of travel is on foot [10]. Distance and travel time to primary health centers (PHC) in these areas are known drivers of care utilization, showing consistent negative impacts on the use of prenatal, perinatal and obstetric care for women [4, 11–15]; child vaccination coverage and pediatric health utilization [16–19]; voluntary enrolment in health insurances [2]; and rates of diagnosis or treatment for tuberculosis, malaria, and HIV [20–24]. In fact, the use of primary care tends to fall exponentially as distance from health facilities rises, a phenomenon known as “distance decay” [16, 23, 25–27]. Geographical barriers to care can persist even when facility-based HSS activities are in place, making these approaches insufficient to reach full population coverage of primary care services [28–33]. Therefore, to optimize both facility-based and community-based strategies towards the realization of UHC, a much deeper understanding of geographical accessibility to PHC and community health sites (CHS) is necessary in contexts undergoing HSS efforts.

Geographical access to health care has been previously characterized in other contexts using a variety of methods [13, 34–36], aimed at estimating distance or travel time to reach health facilities for populations [17, 37]. A common approach consists of estimating travel time through population surveys, but this method is resource-intensive and prone to biases related to how people subjectively measure time [23, 26, 34]. An alternative approach is the use of geomatics with available geographic information [38–40], either estimating Euclidean distance (“as the crow flies”) or using more precise algorithms that account for terrain characteristics and road networks through friction surfaces [35, 38, 41, 42]. To increase the adoption of geographic accessibility analyses into health planning, the WHO integrated those algorithms into an easy to use, freely available tool (“AccessMod”) that includes multiple functionalities [43, 44]. However, methods that rely on friction surfaces represent only a “best guess” of the routes people use in areas with poor road infrastructure and of the speed at which people travel. For geographical accessibility analyses to be precise enough for local use by program managers and health workers, reliable information on footpath networks needs to be combined with context-specific estimates of travel speed, and integrated via e-health tools. This can inform the design and implementation of geographically targeted interventions that balance facility-based, community health, and outreach strategies in order to maximize population access to primary care.

Such approaches are particularly needed in Madagascar, a country with one of the least funded health systems in the world [45]. In 2014, Madagascar had less than 3 clinicians (doctors, nurses and midwives) per 10,000 people [46], with a lower concentration in rural areas, where over three quarters of the population live [47]. Access to health care is particularly low for populations living more than 5 km away from a PHC, putting them at higher risk for early childhood mortality [48, 49]. In 2014, the Madagascar’s Ministry of Health (MoH) and the nongovernmental organization PIVOT partnered to strengthen the public health system in the rural district of Ifanadiana, with the aim of attaining UHC and set a model for the country. Despite rapid improvements observed in accessibility and health conditions [50, 51], initial analyses suggested that health gains were concentrated in close proximity to health centers [51]. Here, we aimed to develop very precise, context-specific estimates of geographic accessibility to care in Ifanadiana district to help with the design and implementation of interventions that improve access for remote populations. We mapped all buildings and footpaths in the district to accurately estimate the shortest routes to health facilities, and we parametrized travel time estimations with hundreds of hours of fieldwork and remote sensing analyses. We integrated all this information into accessible e-health tools for use by PIVOT, MoH and other local partners.

## Materials and methods

### Study area

The study area is Ifanadiana, a rural health district located 444 km southeast of Antananarivo. The district has an area of 3975 sq. km and is characterized by a mountainous landscape. The district’s health system is comprised of one hospital (CHRD II), 21 PHC facilities and 195 CHS where CHWs provide consultations for children under 5 years and reproductive women; these may be their homes or a designated structure. There is only one paved road crossing the district from West to East (national road RN25) and through Ranomafana National Park. Additionally, there are two non-paved axes connecting the main towns in the North and South of the District, which are partly accessible by 4WD vehicles or all-terrain motorcycles. Most villages in the District are connected to each other by small paths only accessible by foot. High rates of extreme poverty, geographical barriers, and unreliable health services were associated with very limited access to health care in the district in 2014, which was substantially lower than average estimates for Madagascar [49, 52]. Since then, the NGO PIVOT has worked in Ifanadiana in partnership with the Ministry of Health to create a “model district”, so that the experience in this district can help improve national strategies and health policies throughout the country. The intervention included the removal of most point-of-service payments as well as improved facility readiness and clinical programs at all levels of care (i.e. hospital, health centers, and community health). In particular, Ifanadiana is one of the first districts to officially pilot the national policy on UHC, which aims to ensure access to quality healthcare for all through strengthened health systems and a reduction of point-of-care fees. Moreover, PIVOT is piloting alternative, professionalized, models of community health through enhanced supervision by certified nurses, building infrastructure for CHSs in partnership with local communities, and implementing proactive community case management. The work described in this study was in support of these two major initiatives.

### Data collection

#### Participatory mapping with OpenStreetMap

Detailed, freely available data on footpath networks and villages in rural areas of the developing world are necessary to obtain precise routes for accessing care, but this information is largely absent. To fill this gap, we carried out photo-interpretation using very high spatial resolution satellite images in OpenStreetMap (OSM), a collaborative mapping project with tools for drawing roads, houses, and land use contours among others [53]. For this, we collaborated with the Humanitarian OpenStreetMap Team (HOTOSM) [54], an organization that promotes collaborative mapping projects on the OpenStreetMap platform for humanitarian purposes through a dedicated interface and network to a large online community. The district was divided into 3508 tasks of 1 sq. km each. Mapping of each task was done in a two-stage process. First, one or several individuals mapped all paths, roads, buildings, and residential areas (defined as groups of 4 or more buildings) within a particular task. This was done on OSM using Digital Globe Standard imagery for background (30–60 cm spatial resolution), and Bing maps imagery (up to 30 cm spatial resolution) as backup when cloud cover in Digital Globe images prevented their use for mapping. After the task was marked as mapped, it was available for validation by a separate person. The validation stage, which uses the same tools as the mapping stage, allowed making all necessary corrections of each task in order to ensure the consistency and quality of the mapping. After the completion of this mapping in HOTOSM, we carried out an additional mapping of the hydrographic network (streams and rivers) and rice fields, following the same protocol. OSM mapping of Ifanadiana district was achieved in 8 months with the collaboration of 103 participants. To increase participation in the mapping project, we organized 5 “mapping parties” with local universities and OSM groups in both Madagascar and La Reunion. Despite it being a collaborative project and published in the HOTOSM Task Manager, we had few spontaneous contributions and 5 people from our research team mapped 73.6% of the overall project. The geographic data mapped in OSM is now freely accessible to any user and can be queried on QGIS [55] via the QuickOSM plugin, which we used here for retrieving the data for our analyses.

#### Recording travel time on the field

Most travel in Ifanadiana district is done by foot due the minimal transportation infrastructure and steep terrain. To obtain context-specific estimates of travel speed by foot according to terrain characteristics, we recorded GPS data from 168 walking routes across 10 out of the 15 communes of Ifanadiana district between September 2018 and April 2019 (Additional file 1). We collected two types of routes: 1) routes from field expeditions of PIVOT’s community team staff during CHW supervisions, and 2) routes specifically recorded for this project to obtain a larger sample size and wider representation of terrain characteristics, collected by representatives of the PIVOT research team and by the local population. We recorded these tracks using Samsung Tab A10 tablets and the Android app “OsmAnd” version 3.0.2 [56]. OsmAnd is a free map and navigation app based on the OSM database. For each trip, we recorded via OsmAnd the GPS location, time and altitude every 10 s.

### Satellite imagery and remote sensing

We complemented the mapping work in OSM, which provides some elements of land use, with remote sensing analyses of satellite images to identify forests, water bodies and savanna land uses. For this, we used free Sentinel-2 images (level-2A) from August 18, 2018, which were orthorectified, provided Top Of Canopy (TOC) reflectance, and had a 10 m spatial resolution. We used the Dzetsaka plugin for semi-automatic classification in QGIS [57]. First, we manually outlined over fifty polygons representing regions of interest (ROI) for each of the three classes (forests, water bodies and savanna). We then ran random forest algorithms, which have good performance in the classification of remotely sensed data, with good accuracy [58]. The random forest model calculates a response variable by creating many different decision trees and then allocating each multi-layered pixel down each decision tree. The response is then determined by evaluating the responses from all trees. The class that is predicted the most is the class that is assigned to the object. Forty percent of the image’s pixels were used for validation. Second, we used the model to predict values for the whole satellite image in order to obtain a classified image. The last step of the process consisted of a post-classification, where smaller clusters of areas under 10,000 sq. m were removed and replaced by the pixel value of the largest neighbor polygon. This process was done to improve the quality of the classification product. Finally, we merged these supervised classifications with the two thematic classes obtained earlier through OSM (residential areas and rice fields). We validated the land use map by recording 62 control points on the field during four expeditions across the district and we completed these observations by identifying 254 points through Google Earth. We finally computed a confusion matrix to compare observed and classified values by class.

In addition to land cover, we obtained elevation and precipitation data from remotely sensed data. We downloaded the Shuttle Radar Topography Mission (SRTM) Digital Elevation Model (DEM) from the United States Geological Survey (USGS, [59]), which gives elevation with a 30 m ground resolution. We also acquired precipitation estimates from the NASA Prediction of Worldwide Energy Resources (POWER) Project [60], with a spatial resolution of 0.5 * 0.5 degrees.

### Estimation of shortest path distance

**Fig. 1:**
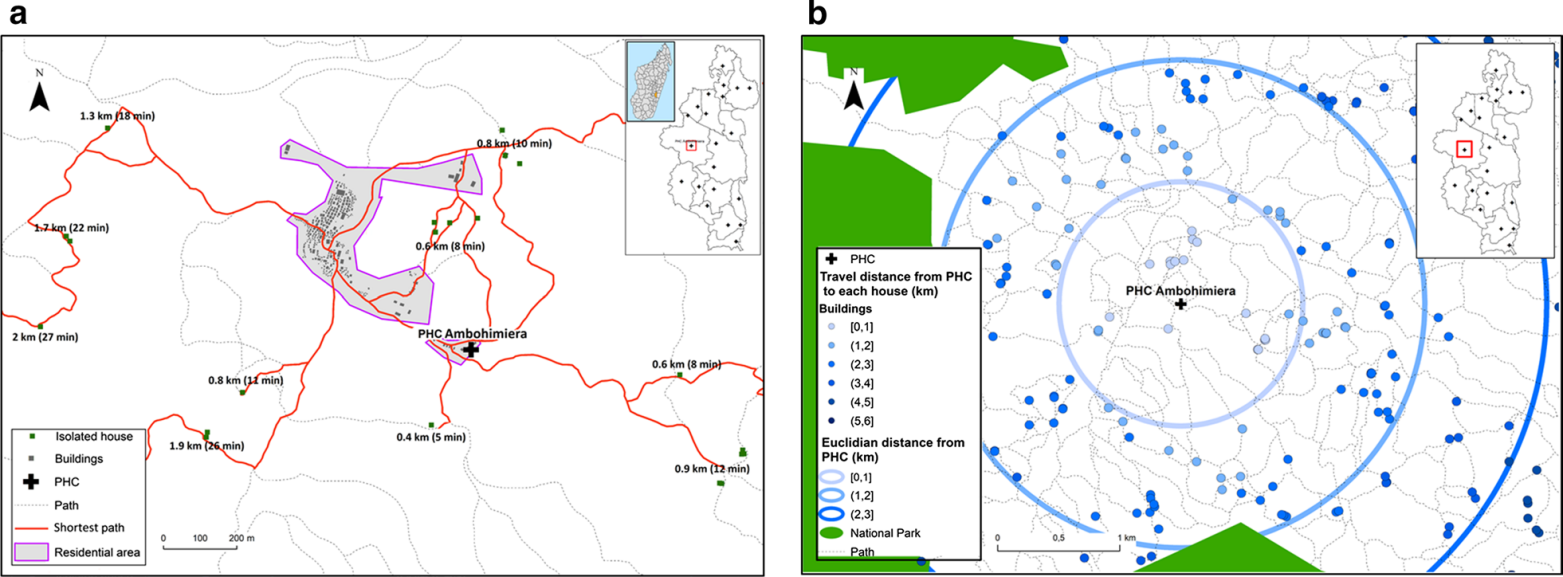
Estimation of the shortest paths from a building to join the PHC. **a** Shows an illustrative example of shortest paths obtained thought OSRM, with building values for travel distance and time to reach one of the district’s PHC **b** Shows how the travel distance calculated by OSRM improves on typical Euclidian distance estimations, providing more realistic and accurate values by using the footpath network

Once the mapping of all buildings and footpaths was finalized, we used the OSRM software [61] to calculate the distance of the shortest path between each building and the closest health facility, via the R software package “osrm” (Fig. 1). OSRM uses the Dijkstra’s routing Algorithm, which searches iteratively the shortest path from a single node to the destination node in a network. For each building, the shortest path distance to two health facilities were calculated: to the closest PHC and to the closest CHS. While we had the location of all PHCs and the 37 CHSs built with PIVOT support, for CHSs without precise GPS locations (158 out of 195), we assumed that they were located in the main village of the Fokontany (“*chef lieu*”), as indicated in national policies for community health. In addition to the distance values, the actual shortest path was saved as a vector file (shapefile format) for use in travel time estimations (next section). Finally, we interpolated the distance values in the whole district using kriging methods available in ArcGIS to improve visualization of results.

### Estimation of travel speed and time to seek treatment

To obtain precise and context-specific estimates of time to seek treatment, we studied the geographic and climatic factors associated with travel speed in the sample of 168 walking routes collected on the field. For this, travel speed between each pair of points within a GPS track was estimated using the time and GPS location at which each point was taken. Then, we divided the 168 walking routes into 100 m segments and we intersected these segments with the raster datasets to obtain corresponding values for DEM, land cover and rainfall. For this, we used the “st_intersects” function available in PostGIS software. As a result, between each pair of points (N = 57,719) we obtained values for the following explanatory variables: degree of slope, cumulative distance since the beginning of the track, precipitation and land use.

We modelled the impact of each geographic and climatic factor on travel speed using additive models that included a random intercept for each individual track. First, exploratory and univariate analyses were carried out to understand the relationship between each variable and travel speed, including linear and non-linear relationships for slope as well as categorical and numerical variables for land use and cumulative distance (Additional file 2). Cumulative distance since the beginning of the track was categorized following exploratory analysis into 2 groups (0 to 13 km and 13 to 25 km) to reflect the reduction in speed after substantial walking. The land use was converted into a categorical variable that represented the predominant thematic class between each pair points, and a category “mixed” was added when the predominant class represented less than 50%. Slope was included as a non-linear smooth in the additive model. All these explanatory variables had a *p* value under 0.1 and were included in the multivariate analysis. Model fit was estimated via AIC (Akaike information criterion), whereby the model with the lowest AIC was selected. Model validation was carried out to check for normality, homogeneity and independence of residuals.

Using travel speed estimates from the fixed effects of the final multivariate model, travel time was predicted for each of the 41,426 routes obtained through OSRM (two per isolated building or residential area, one to the closest PHC and one to the closest CHS). For this, similarly to the 168 fieldwork routes process, we divided these routes into 100 m segments and intersected each segment with DEM and land use to obtain the same set of explanatory variables. Since rainfall affects travel speed and varies from day to day, for each route we provided a prediction for a scenario without rainfall (minimum time) and with the maximum amount of rainfall recorded during fieldwork (maximum time). Although predictions of travel time for these routes were not validated a posteriori on the ground, the 168 fieldwork routes used to parameterize the model were representative of the larger PHC and CHS route datasets (Additional file 1). As with travel distance, we used kriging methods to interpolate the values of travel for the entire district to improve visualization.

### Comparison of results with existing methods

To assess whether the methods used in our study improved the precision of existing methods used in geographic accessibility modeling, we compared estimates of travel time obtained here with results obtained from Euclidean and friction surface methods. For this, we compared absolute and relative differences for the 168 routes for which we had field information as well as for the 41,426 routes predicted in our analyses. For Euclidean distance we used the R package “stats” and we assumed a constant travel speed of 5 km/h. For friction surfaces, we used the software “AccesMod” together with the district’s digital elevation model, OSM road network and land cover datasets, and we assigned speed values for each class of land cover and road network following recommendations for AccessMod 3.0 available in Ray et al. [43].

### Development of an e-health tool with R Shiny

We developed an online app to facilitate the use by local health staff of the data and results from the study. It consists of a website interface that builds on the estimation methods for distance and travel time in Ifanadiana district presented here, to make the results flexible and easily accessible by program managers and health workers (in French and English). We used the package Shiny [62] for R statistical software. This app is hosted at the PIVOT dashboard website (http://research.pivot-dashboard.org:3838/) for both private and public use.

## Results

### Mapping

A total of 106,171 buildings were mapped. Of these, 65.6% were located in one of the 4925 residential areas, whereas 34.4% were isolated houses. The size of residential areas ranged from 4 to 870 buildings, with an average size of 14. Moreover, 23,726 km of footpaths were mapped, which represent 99.1% of the road network in Ifanadiana district. Only 0.3% of the road network (62 km) are paved secondary roads. To expand available data, we also mapped on OSM the name and location of 707 villages, 21 PHCs (2 of which were recently built), and 37 CHSs built with PIVOT support. This district GIS dataset was substantially more exhaustive than what is available through OSM for the rest of districts in the Vatovavy Fitovinany region, comprising an area of approximately 16,788 sq km (Table 1).

**Table 1:** Comparison of geographic information available on OSM in Ifanadiana district following mapping and the average geographic information available in the other districts of Vatovavy-Fitovinany Region

	Ifanadiana				Distric average^a^ for the rest of Vatovavy-Fitovinany region			
	n	(%)	Length (km)	Area (ha)	n	(%)	Length (km)	Area (ha)
*Residential area*	4925				223			
[0, 20]	4241	86.11			206	92.38		
(20, 40]	371	7.53			9	4.03		
(40, 50]	58	1.18			1	0.45		
(50, 100]	177	3.59			4	1.19		
> 100	78	1.58			3	1.35		
*Buildings*	106,171				6723			
Isolated house	36,539	34.42			5086	75.65		
On residential area	69,632	65.58			1637	24.35		
*Rice field*	17,446			13,436	201			297
*Road networks*								
Path			23,726				400	
Secondary road			62				68	
Tertiary road			130				45	

^a^The average here represents the sum of the number of residential areas, buildings, rice fields and the length of road networks in the other 5 districts of the region (Manakara, Mananjary, Ikongo, Vohipeno, Nosy-Varika) divided by 5

The remote sensing analysis allowed to map 850 sq km of forest, 12 sq km of water bodies and 2967 sq km of savanna (21.4%, 0.30% and 74.6% of the district surface, respectively) at a 10 m resolution (Additional file 3). The kappa index for the land use classification was 0.95 [58].

### Shortest path distance to health facilities

**Fig. 2:**
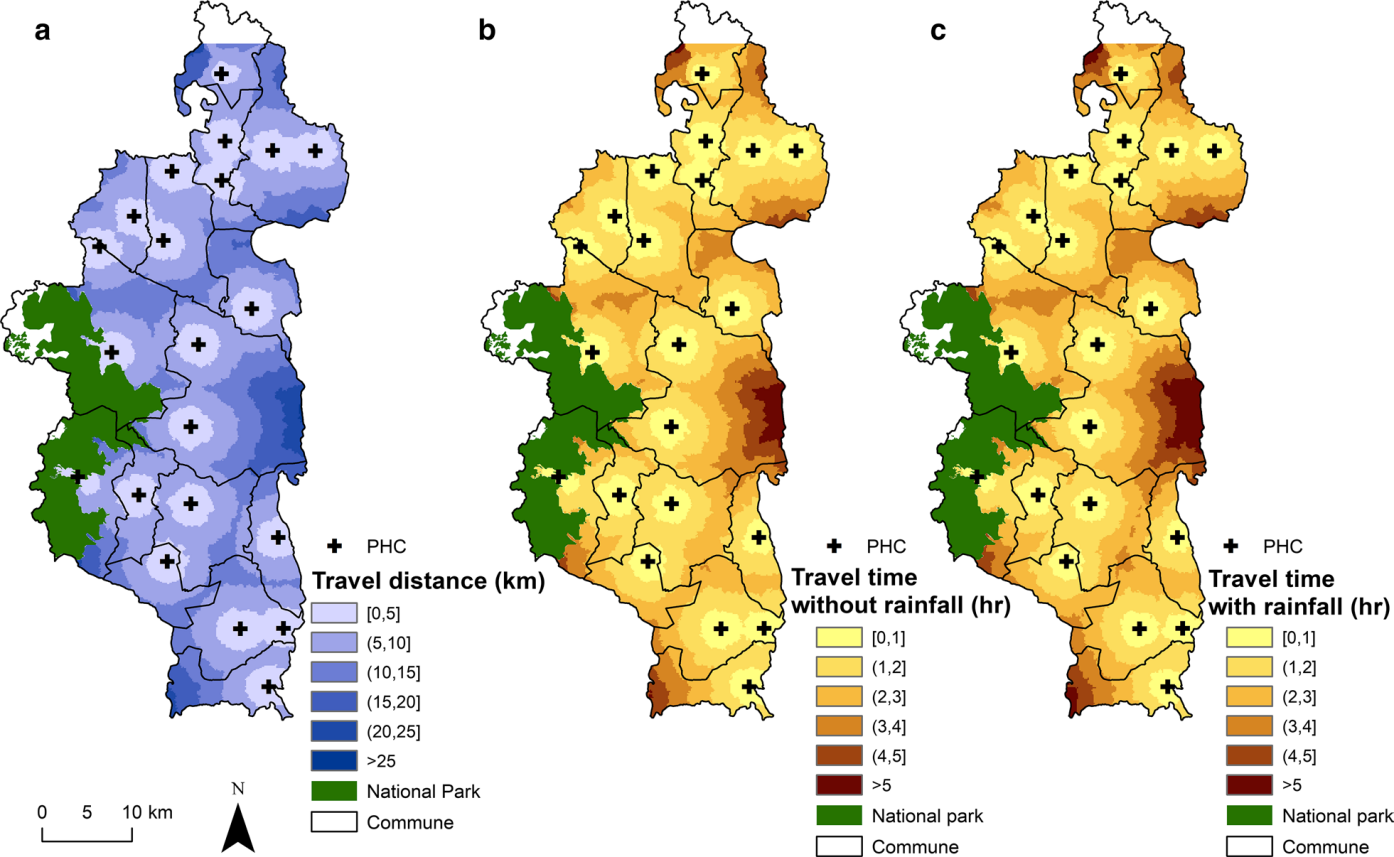
Interpolated distance and travel time between each household and PHCs. **a** Spatial variation in the distance to join the nearest PHC, with shades of blue representing 6 distance classes: 0–5, 5–10, 10–15, 15–20, 20–25 and > 25 km. **b**, **c** Travel time without and with rainfall to join the nearest PHC, with shades of brown representing 6 time classes: 0–1, 1–2, 2–3, 3–4, 4–5 and > 5 h

**Fig. 3:**
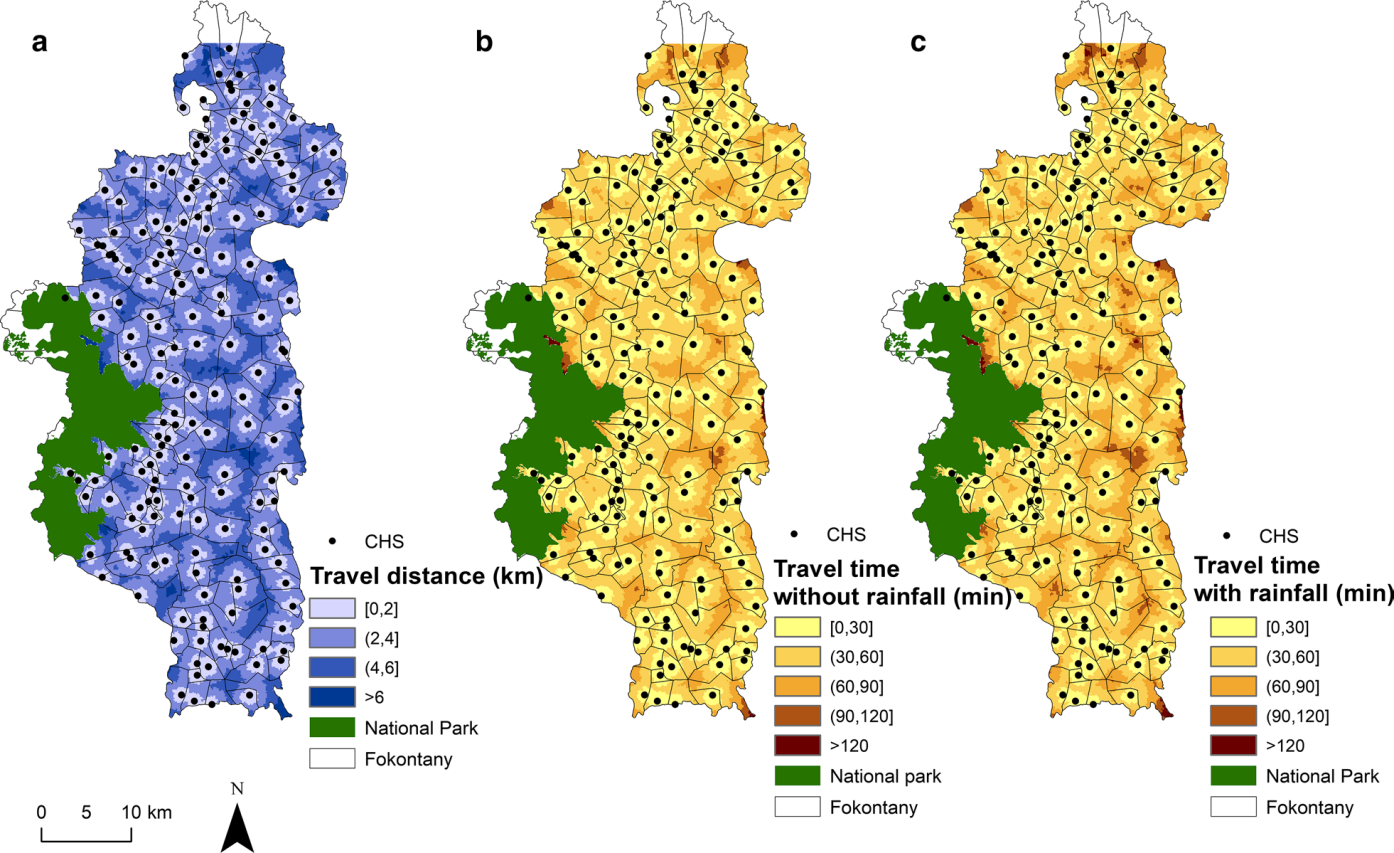
Interpolated distance and travel time between each household and CHSs. **a** Spatial variation in the distance to join the nearest CHS, with shades of blue representing 4 distance classes: 0–2, 2–4, 4–6 and > 6 km. **b**, **c** Travel time without and with rainfall to join the nearest CHS, with shades of brown representing 5 time classes: 0–30, 30–60, 60–90, 90–120 and > 120 min

Travel distance to reach the nearest PHC varies from 0 to 27 km with a district average of 8 km (Additional files 4 and 5). The most remote areas in terms of PHC accessibility are located in the North (Fasintsara), South-West (Ranomafana), and East of the district (Tsaratanana), where populations live further than 20 km from the nearest PHC (Fig. 2). More than two-thirds of the district population live further than 5 km from the closest PHC, with 44.43% living between 5 and 10 km, and 24.67% living between 10 and 20 km (Table 2). Only 8.54% live under 2 km from a PHC. Travel distance to reach the nearest CHS varies from 0 to 13 km, with a district average of 2.69 km (Additional files 4 and 5). In several areas across the district, populations live between 4 and 6 km to the closest CHS (Fig. 3). Overall, less than 5% of the population resides more than 5 km away from a CHS, but half (52.2%) live between 2 and 5 km. Only 43.75% live under 2 km from a CHS (Table 3).

**Table 2:** Distribution of the population in Ifanadiana according to their distance to the nearest health facility

Health facility type	Travel distance (km)	Population (%)
Primary health care center (PHC)	[0, 1]	5.85
	(1, 2]	2.69
	(2, 5]	21.02
	(5, 10]	44.43
	(10, 20]	24.67
	(20, 30]	1.34
Community health site (CHS)	[0, 1]	22.07
	(1, 2]	21.68
	(2, 5]	52.2
	(5, 10]	3.98
	(10, 20]	0.08

**Table 3:** Multivariate analysis of local factors affecting travel speed by foot in Ifanadiana, (linear additive model, with individual track as random intercept)

	Coeff	Std. Error	p
*Intercept (km/h)*	3.27	0.10	< 0.001
*Slope (%)*	Non-linear^a^	-^a^	< 0.001
*Rainfall (every 10 mm)*	− 0.06	0.01	<0.001
*Travel distance (km)*			
[0, 13]	(Ref)		
(13, 22.9]	− 0.38	0.02	< 0.001
*Land cover*			
Mixed	(Ref)		
Water bodies	− 1.32	0.18	< 0.001
Forest	0.01	0.16	0.93
Rice field	− 0.46	0.16	< 0.01
Savanna	− 0.05	0.16	0.75
Residential area	− 0.52	0.16	< 0.01
*Individual*			
Community team staff	(Ref)		
PIVOT research team	1.20	0.03	< 0.001
Local population	1.29	0.03	< 0.001

^a^There was an exponential decrease of speed at higher absolute values of slope (in percent). Non-linear smooth and confidence intervals are shown in Additional file 6

### Time to seek care under different climatic conditions

Statistical analyses from a sample of 168 walking routes in Ifanadiana District (871 km, Additional file 2) allowed us to identify the most relevant determinants of travel speed for local populations and health workers. Overall, there was wide variation in speed between individual tracks, with 39.45% of the variation explained by the random effects. Travel speed was reduced by 0.38 km/h after the first 13 km. In terms of terrain characteristics, slope was the most important determinant of travel speed, which decreased exponentially with absolute values of slope (whether positive or negative, see Additional file 6). Walking through a water body, a rice field, or a residential area were associated with slower travel speeds, whereas walking through savanna, forest or a mixed land use were associated with faster speed. Finally, rainfall had a small negative effect on travel speed, where an increase in 10 mm of rainfall was associated with a reduction of 0.06 km/h in speed.

Using predictions of travel speed for the 41,426 routes obtained through OSRM we found that time to seek care at a PHC varies between 0.49 min and 7 h (one way) under dry conditions (average of 111 min) and between 0.51 min and 7.5 h during rainy conditions (average 121 min; Additional files 3 and 4). The time difference between rainy and dry conditions was most relevant (over 20 min) for areas located further than 15 km to the PHC. Overall, only 21.6% of the population can join a PHC within an hour under rainy conditions and 58.1% within 2 h. This percentage increases under dry conditions to 24.3% of the population being able to reach the PHC within an hour, and 63.2% within 2 h.

**Fig. 4:**
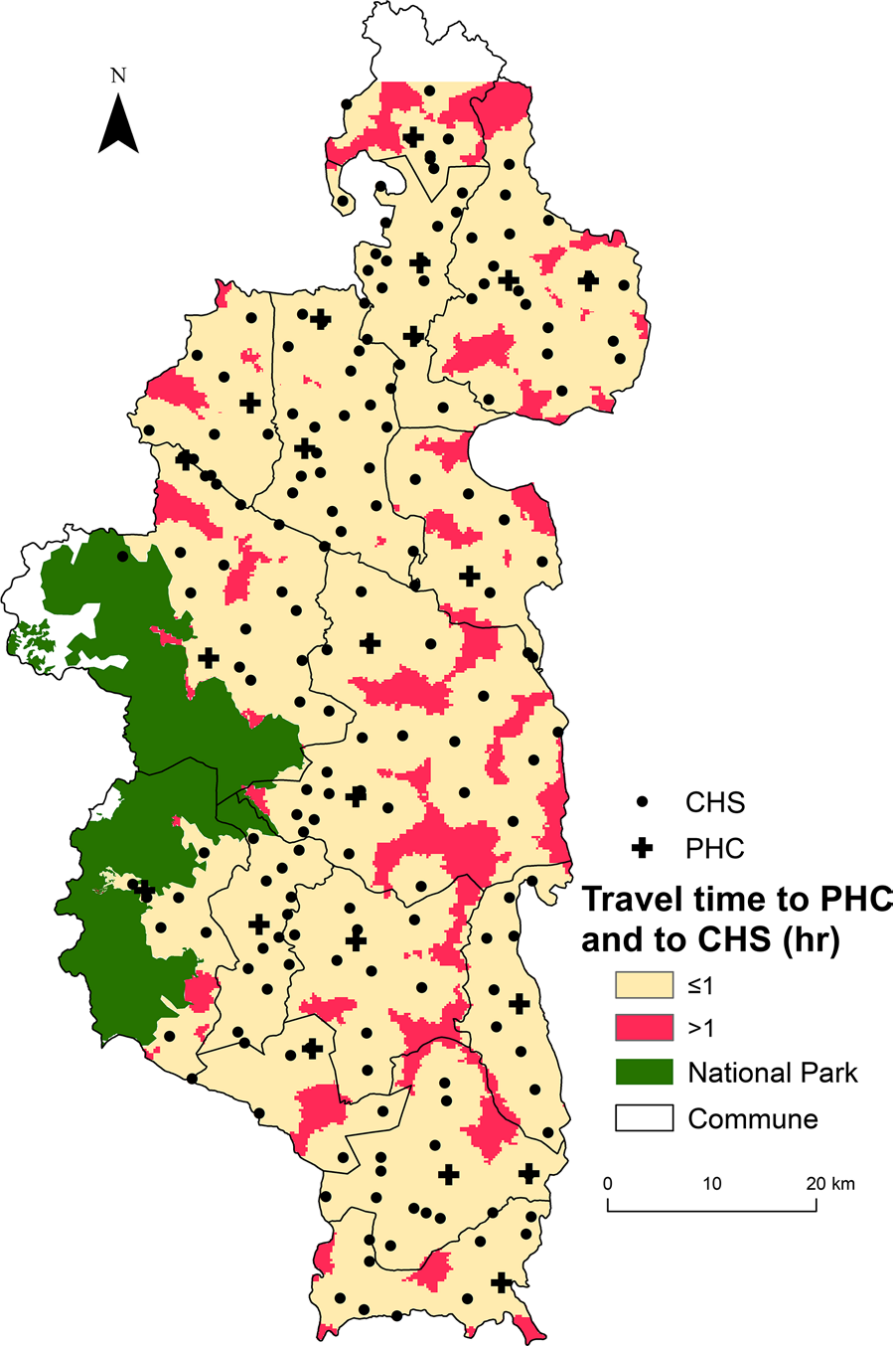
Distribution of vulnerable populations with poor geographic access to both PHC and CHS. It shows the spatial variation in the travel time to reach any type of primary care facility (both PHC and CHS). Areas less than 1 h away are shown in light brown and those more than 1 h away in red

Travel time to reach a CHS varies between 0 and 150 min under dry conditions, and up to 165 min under rainy conditions (Additional files 3 and 4). For areas located further than 5 km from a CHS, the difference between dry and rainy conditions can be of 15 min. Overall, 40% of the population lives within 30 min from a CHS, and 85.12% lives within 1 h under rainy conditions (Table 4). This percentage increases under dry conditions to 44% and 89.83% for populations living within 30 min and 1 h to a CHS, respectively. Small areas all across Ifanadiana had populations that lived further than 1 h from a CHS (Fig. 3), and most of these populations lived further than 1 h from any type of primary health facility, whether a PHC or a CHS (Fig. 4).

**Table 4:** Distribution of the population in Ifanadiana according to their travel time to the nearest health facility

Health facility type	Travel time (minutes)	Population (%)	
		If time estimation without rainfall	If time estimation with rainfall
Primary health care center (PHC)	[0, 30]	9.76	8.85
	(30, 60]	14.49	12.77
	(60, 120]	38.90	36.5
	(120, 180]	25.20	26.19
	(180, 240]	7.97	10.54
	> 240	3.66	5.14
Community health site (CHS)	[0, 30]	44.24	39.98
	(30, 60]	45.59	45.14
	(60, 120]	9.95	14.59
	(120, 180]	1.21	0.27
	(180, 240]		0.03

### Comparison of travel distance and time with commonly used geographic access modeling methods

Comparisons of estimates with other commonly used methods in geographical access modeling showed that methods presented here improved the precision of travel time in Ifanadiana district (Table 5 and Additional file 7). Using Euclidean distance and an average speed of 5 km/h resulted in a relative difference with values obtained during fieldwork of 40.5% (31.22 min). Using friction surfaces through AccessMod the difference with fieldwork values was 22.57 min (29.81%) on average. While Euclidean methods consistently underestimated travel distance and time in this sample of routes, the friction surface method resulted in more variable results (Additional file 7). In contrast, predictions using OSRM and our statistical model resulted in smaller relative differences of 4.27% (3.6 min).

**Table 5:** Comparison of travel time with Euclidean and friction surfaces methods

Time estimation method^a^	Sample of fieldwork routes (N = 168)		All routes (N = 41,426)			
	Absolute difference (minutes)	Relative difference (%)	PHC		CHS	
			Absolute difference (minutes)	Relative difference (%)	Absolute difference (minutes)	Relative difference (%)
Fieldwork	Reference	Reference	–	–	–	–
OSRM + statistical model	3.6	4.27	Reference	Reference	Reference	Reference
Euclidian distance + 5 km/h speed	31.22	40.5	47.54	36.89	13.27	34.47
Friction surfaces + custom speed values	22.57	29.81	26.31	19.88	7.86	25.35

^a^Absolute values are used to allow for consistent average estimations. The distribution of differences (with signs to reflect underestimation or overestimation) is shown in Additional file 7

When we extended this comparison to all 41,426 routes between households and health facilities, using our estimates as a reference, Euclidean methods resulted in a relative difference of over 30% (48 and 13 min for PHC and CHS, respectively). Differences in travel time with friction surface methods were lower, at 20% for PHC (26.31 min) and 25% for CHS (7.88 min). As with the fieldwork sample, Euclidian methods consistently underestimated travel time in PHC and CHS routes. In contrast, friction surfaces overestimated travel time in most of these routes when compared with our methods (Additional file 7).

### e-Health tools for geographical accessibility to care

**Fig. 5:**
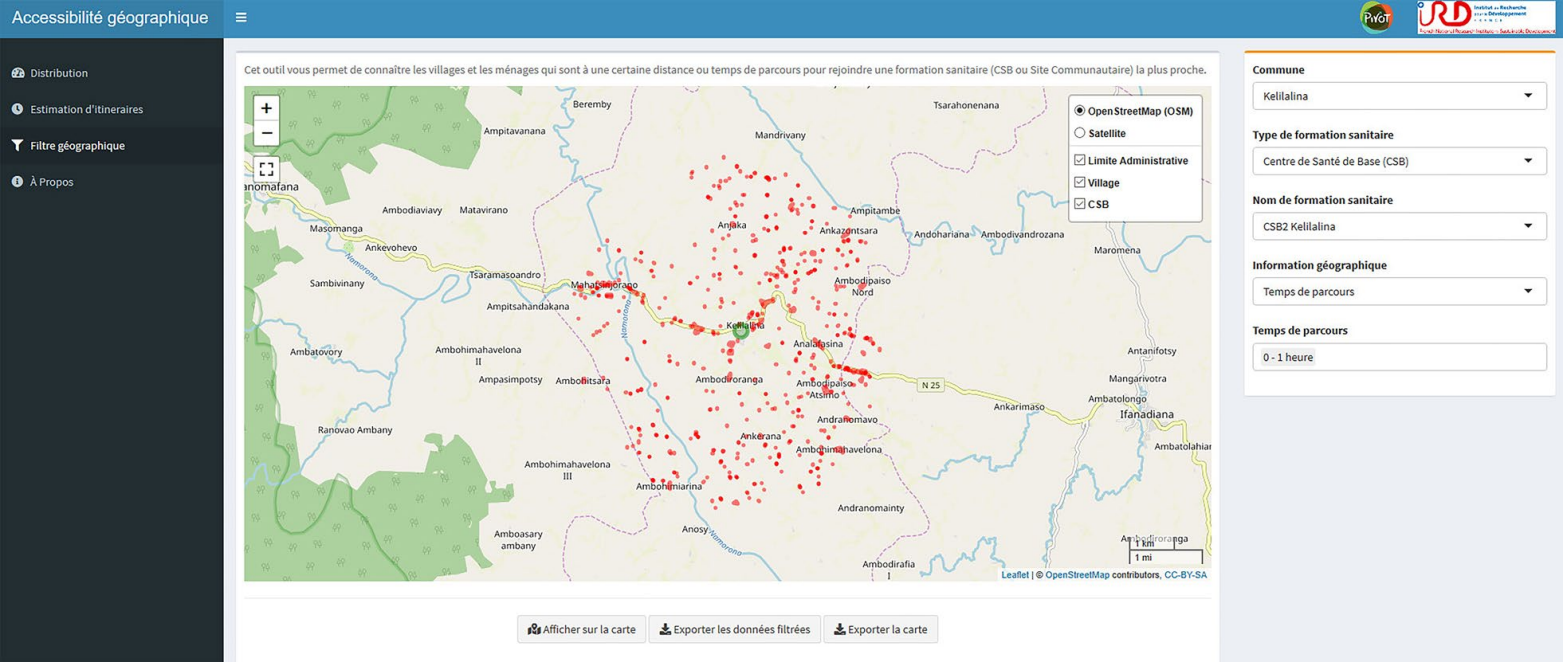
Shiny app for operational use by local health actors. Illustrative example of the interface, showing in a map all the residential areas and isolated houses (red polygons) located between 0 and 1 h from a selected PHC (green circle)

The web interface created for health workers and program managers (Fig. 5) allows visualization of key information for the planning and implementation of health programs such as (1) areas with the lowest accessibility to health care based on shortest path distance and travel time, (2) the percentage of the population in each commune and fokontany that are at a particular distance and travel time to the nearest health facility, (3) a tool to estimate shortest path routes for field expeditions and community health work with either a satellite or OSM background, and 4) the geographic distribution of residential areas and isolated households that are within a certain distance or time from a selected health facility. In addition to the web interface, which can be accessed by phone or computer but requires an internet connection, field workers can get accurate directions without internet access. For this, they can use the free Android app “OsmAnd” on a smartphone or tablet, as the full footpath network and all residential areas have been mapped on OSM in this project, and this app only needs the phone’s GPS and OSM maps stored locally.

## Discussion

Despite a renewed global commitment in recent years to UHC and community health as ways to increase financial and geographical access to primary care, half of the world’s population continues to lack access to essential health services [1]. Distance to health facilities remains one of the main barriers to accessing care in rural areas of the developing world, where transport infrastructure is deficient, population density is low and health facilities are sparse. Although methods and tools exist to model geographic accessibility, there is a critical lack of basic geographic information in low-resource rural settings that prevents their widespread use to optimize the implementation of local health programs. Using a health district of Madagascar as case study, we show how the combination of participatory mapping, fieldwork and remote sensing can provide very precise estimates of distance and time to seek treatment at health facilities that can be used to inform health system design and policy, improve management, and support health workers. We found that over three quarters of the district population lived more than 1 h from a PHC. Moreover, we identified several areas where the nearest PHC was further than 5 h away, and vulnerable communities in most parts of the district with poor geographical access (over 1 h travel) to both PHCs and CHSs.

A travel time of 1 or 2 h to health services is a generally accepted threshold of poor accessibility to health services, delaying or preventing health seeking behaviors that can result in severe health consequences [22, 26, 38, 63, 64]. While there are no estimates of geographical access to primary care for sub-Saharan Africa, only 11–27% of the population were estimated to live further than 1 h from a hospital with surgical capabilities [64]. National and sub-national level studies focusing on Emergency Obstetric and Neonatal Care (EmONC) found slightly higher results, with 30% of the women in two regions of Ethiopia [38] and 34% in Ghana living further than 2 h from EmONC facilities [26]. In contrast, we found that three quarters of the population in Ifanadiana district (76%) lived more than 1 h from a PHC (without surgical capabilities) and forty percent lived further than 2 h, with a larger percentage in the rainy season. Thus, our study suggests that geographic access to primary care in rural Madagascar is significantly lower than estimated elsewhere. If these geographic challenges are representative of other low-resource rural settings, our results could have important implications for UHC policies. Indeed, UHC policies tend to increase financial access to primary care by reducing point-of-service payments (e.g. health insurance), but geographic barriers to care can persist even when fees are removed [28–33], making them insufficient unless complementary policies targeting geographic accessibility are in place. Experiences from other countries suggest that health reforms focused on improving geographical accessibility to primary care can achieve significant results for the most disadvantaged populations [65].

In particular, community health is considered a key solution for reducing geographical inequities in access to care in developing countries. While the WHO has released comprehensive guidelines to optimize community health programs [10], there is a critical lack of evidence around the geographical accessibility to community health workers. In Madagascar, the national policy for community health establishes that two CHWs should be appointed in every Fokontany, with no regard to the size or spread of the population in the Fokontany. Here we show that over half of the district’s population had to walk over 30 min to reach a CHS, and 10 to 15% had to walk over 1 h depending on the season. This suggests that gaps exist even in the geographical coverage of community health programs and opens new questions around how to measure and optimize access to community health for rural populations, based on a deep understanding of their geographical context. A shift towards proactive community care programs could help ensure the provision of essential services to populations despite their distance to a CHS, improving health conditions [66]. Alternatively, in settings that rely on standard community programs, adapting the CHW workforce to the demographic and geographic characteristics of the population catchment could help improve their geographical reach.

In this study, we estimated distance to PHC using a mapping of about 23,000 km of footpaths and over 100,000 buildings, and we parametrized travel time with hundreds of hours of fieldwork, obtaining precise and context-specific accessibility estimates for every community in our district. This approach overcomes many of the challenges that studies of geographical accessibility commonly face. Despite the increasing use of geographical information systems and spatial analyses to better understand health care access in developing countries, there are still important gaps in its application. Studies in high income countries provide accurate estimates of geographical access to services due to the wider availability of electronic health information systems and GIS data, as well as good transport infrastructure [63, 67–70]. In contrast, studies in developing countries typically use either Euclidean distances to obtain a basic measure of distance, or road networks in combination with friction surfaces to obtain estimates of travel time to PHC because the real network of footpaths is rarely available [21, 27, 41]. Given that most travel in rural areas is done by foot, inaccuracies from such models can be quite important [22, 71]. In addition, while travel time can strongly depend on contextual factors, researchers use either constant values for travel speed [20, 26] or values for different geographic features inputted from other contexts [22]. In our context, comparisons of our estimates with these methods showed average differences in travel time of 30–35% for Euclidian distances and 20–25% for friction surfaces.

Our approach could be replicated in future studies of geographical accessibility to care in rural areas of the developing world, where very precise estimates of distance and time to seek treatment are required for local use by decision-makers, program managers and health workers. Mapping on OSM is simple, the information entered is accessible to anyone for download or for use into mobile apps, and the geographic database can be regularly updated or further expanded by the online community. Although in our case most of the work through HOTOSM was done by several mappers involved in the project and took several months, this type of participative and collaborative mapping approach can allow for rapid crowdsourced mapping of large geographical areas when the mapping community is heavily mobilized [72]. In addition, recent developments in artificial intelligence such as the Maxar-Digital Globe building footprints, or the RapiD tool developed by Facebook for road networks could greatly accelerate mapping in low-resource settings, making some of the methods presented here more easily scalable.

Besides the broader policy implications for community health and UHC outlined above, our study provided several programmatic insights to improve health care access in Ifanadiana. In the last 3 years, two PHCs have been built in the extreme North and South of Ifanadiana where geographical access was very low, reducing the percentage of population that have to walk 2–5 h (Additional file 8). Given results outlined in Fig. 2, adding three new facilities to the 21 existing PHCs in the areas of the district with the lowest geographical access (East, West and Southwest) would nearly eliminate the need for populations to walk 4 h or more each way to a PHC. In addition, several programs are being implemented to improve geographical access to primary care for remote populations, including the construction of maternal waiting homes near PHCs for expecting mothers and the provision of health services by teams of nurses during regular expeditions. For community health, geographic barriers to reach CHSs observed here motivated a pilot program on proactive community case management, which will be progressively scaled up if successful. The e-health tools developed as part of the study will allow program managers to prioritize and plan these community and outreach activities for the most remote populations, while CHWs and field teams will be able to obtain accurate directions anywhere in the district for implementation of such activities. Future operational research should assess the uptake and impact of such tools on health care delivery.

Our study had several limitations. First, dates of the satellite images used for the OSM mapping were not available, which could pose an issue if images did not reflect current conditions, but subsequent fieldwork confirmed that OSM maps (buildings, paths, etc.) were quite accurate. Second, we only considered a model of travel time by foot, which could lead to biases if part of the population travels to PHCs by vehicle. Other studies have indeed estimated travel time by vehicle in settings with good road networks [34, 69], or by both foot and vehicle in low-resource settings [35]. In our context, less than 3% of the population of Ifanadiana has a vehicle, and there is only one paved road (< 1% of the road network) where public transportation is available [73]. Third, we infer the proportion of the population living at a particular distance or travel time based on a full mapping of the district’s buildings from high resolution satellite imagery instead of an actual census. Since some of these buildings do not represent inhabited households (e.g. administrative buildings, shops, etc.), this approach could have led to biases if the distribution of non-households was distributed heterogeneously across the district.

While we provide locally parametrized estimations of travel time for Ifanadiana, the geographic and climatic variables assessed had modest effects on travel speed. Overall, 40% of travel speed in our model was explained by the random effects, which suggest that innate differences in walking speed between individuals might be one of the most relevant determinants of travel time. Regarding climate, our estimates under scenarios with and without rainfall differed only by up to 20 min at distances of over 15 km. Although the district has a tropical climate with periods of heavy rains, track GPS recordings of field expeditions were collected during relatively low rainfall periods (up to 47.5 mm per day), which could explain the small impact of rainfall on travel speed. In addition, the coarse resolution of available rainfall data for our area (0.5 degrees) could have biased the estimates of rainfall and impacted its influence in our model. Rainfall could also lead to the isolation of areas during the rainy season due to flooding or a rise in river water levels [35], but we could not estimate these effects in our setting from remote sensing data. In terms of land cover, only water surfaces and rice fields were associated with lower speed values, and removing land cover effects resulted in minimal changes in our predictions of travel time (Additional file 9). Finally, recordings were done by health workers and community members, so our estimations represent local travel time for healthy individuals. Other groups such as ill individuals, pregnant women or the elderly will likely take longer to reach health facilities, and factors such as break time during a route were not considered, which could be particularly relevant for those who walk for 4 to 8 h. As a result, values for maximum time to seek treatment at health facilities presented here are probably an underestimation of the real time spent by certain groups or under certain weather conditions.

## Conclusion

This study advances methods to improve geographical accessibility modeling to determine with high precision the shortest paths and travel time between every household and the nearest primary care facilities, so that the results can be context-specific and operationally actionable by local health actors. Integrating the complete mapping of the district with fieldwork and remote sensing analyses into accessible e-health tools may result in better strategic and operational refinement of programs by the MoH and local health partners. The role of such approaches could be transformative for reaching the goal of “health care access for all” in areas where, like in Ifanadiana, the majority of the population face significant challenges to reach primary care facilities and even CHSs.

## Supplementary information

**Additional file 1.** Representability of fieldwork routes relative to the total number of routes estimated in geographic accessibility analyses.

**Additional file 2.** Explanatory analyses of all variables, with 3 quantitative variables (slope, distance and rainfall) and 2 qualitative variables (land cover, individual type).

**Additional file 3.** Map of land cover in Ifanadiana district, 2018, at a 10 m resolution following remote sensing analyses.

**Additional file 4.** Histogram of travel distance and time to PHC and CHS.

**Additional file 5.** Cumulative percentage of the population at each distance and time to seek treatment from the closest PHC and CHS.

**Additional file 6.** Non-linear smooth and 95% confidence intervals of the effect of slope change (%, absolute values) on travel speed in the multivariate GAM model presented in Table 3.

**Additional file 7.** Comparison of travel time with other commonly used methods in geographic access modeling.

**Additional file 8.** Comparison of travel time for the population of Ifanadiana between a scenario with 19 PHCs and a scenario with 21 PHCs, after the construction of two new PHCs in 2016 and 2018.

**Additional file 9.** Histogram of relative difference in travel time estimations for PHC and CHS routes between a statistical model with land cover and without land cover.

## Data Availability

Data are available on OpenStreetMap (https://www.openstreetmap.org) and on the Shinny app described in this study (http://research.pivot-dashboard.org:3838/).

## Abbreviations

CHS: Community health site
CHW: Community health worker
DEM: Digital elevation model
EmONC: Emergency obstetric and neonatal care
GPS: Global positioning system
HOTOSM: Humanitarian OpenStreetMap Team
HSS: Health system strengthening
MoH: Ministry of Health
OSM: OpenStreetMap
OSMR: open source routing machine
PHC: Primary health care center, POWER: prediction of worldwide energy resources
SRTM: Shuttle radar topography mission
UHC: Universal health coverage
