# Supplementary material for: Improving geographical accessibility modeling for operational use by local health actors
Source: Int J Health Geogr. 2020 Jul 6;19:27. doi: 10.1186/s12942-020-00220-6 (PMC7339519; doi:10.1186/s12942-020-00220-6)
Supplement: Supplementary file 1 — Additional file 1. Representability of fieldwork routes relative to the total number of routes estimated in geographic accessibility analyses. [file 12942_2020_220_MOESM1_ESM.docx]

**Additional file 1:** Representability of fieldwork routes relative to the total number of routes estimated in geographic accessibility analyses

| **Variable** | **Categories** | **Fieldwork routes (N=168)** | **PHC routes (N=41,426)** | | **CHS routes (N=41,426)** | **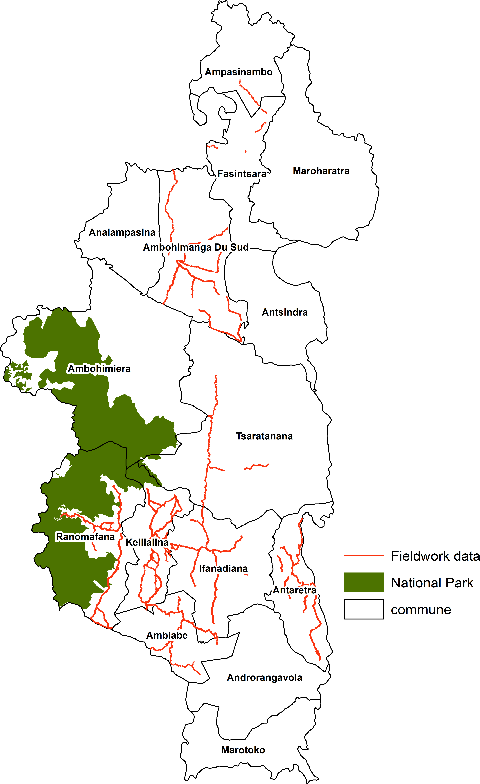** |
| --- | --- | --- | --- | --- | --- | --- |
| Land cover (%) | Water bodies | 0.01 (0.22) | 0.10 (0.14) | 0.003(0.14) | |  |
|  | Forest | 0.18 (4.06) | 0.19 (2.70) | 0.08 (3.49) | |  |
|  | Mixed | 0.03 (0.63) | 0.15 (2.02) | 0.05 (1.96) | |  |
|  | Rice field | 0.25 (5.52) | 0.21 (2.86) | 0.06 (2.47) | |  |
|  | Savanna | 3.75 (83.11) | 6.36 (88.66) | 2.13 (90.08) | |  |
|  | Residential area | 0.29 (6.46) | 0.26 (3.61) | 0.04 (1.85) | |  |
| Slope (%) | [0,30] | 4.47 (99.09) | 6.87 (95.73) | 2.27 (95.62) | |  |
|  | (30,60] | 0.03 (0.76) | 0.30 (4.17) | 0.10 (4.27) | |  |
|  | (60,100] | 0.006 (0.15) | 0.007 (0.10) | 0.002 (0.10 | |  |
| Distance (km) | [0,13] | 3.71 (82.28) | 5.78 (66.55) | 2.69 (99.79) | |  |
|  | (13-25] | 0.8 (17.72) | 2.88 (33.08) | 0.005 (0.21) | |  |
|  | >25 | - | 0.03 (0.39) | - | |  |

Average values in km (and % between brackets)

The average value of each category is the total distance of all the route portions that contain that category divided by the total number of routes (168 or 41,426)

For each variable, the % is the average value of each category divided by the sum of average values of all categories within that variable
