# Supplementary material for: Improving geographical accessibility modeling for operational use by local health actors
Source: Int J Health Geogr. 2020 Jul 6;19:27. doi: 10.1186/s12942-020-00220-6 (PMC7339519; doi:10.1186/s12942-020-00220-6)
Supplement: Supplementary file 2 — Additional file 2. Explanatory analyses of all variables, with 3 quantitative variables (slope, distance and rainfall) and 2 qualitative variables (land cover, individual type). [file 12942_2020_220_MOESM2_ESM.docx]

**Additional file 2:** Explanatory analyses of all variables with 3 quantitative variables (slope, distance and rainfall) and 2 qualitative variables (land cover, individual type). A) represents frequency histogram of each variables B) Boxplot of the travel speed difference for each variables C) linear and nonlinear relationship with travel speed

|  | **A** | **B** | **C** |
| --- | --- | --- | --- |
| **Speed** | 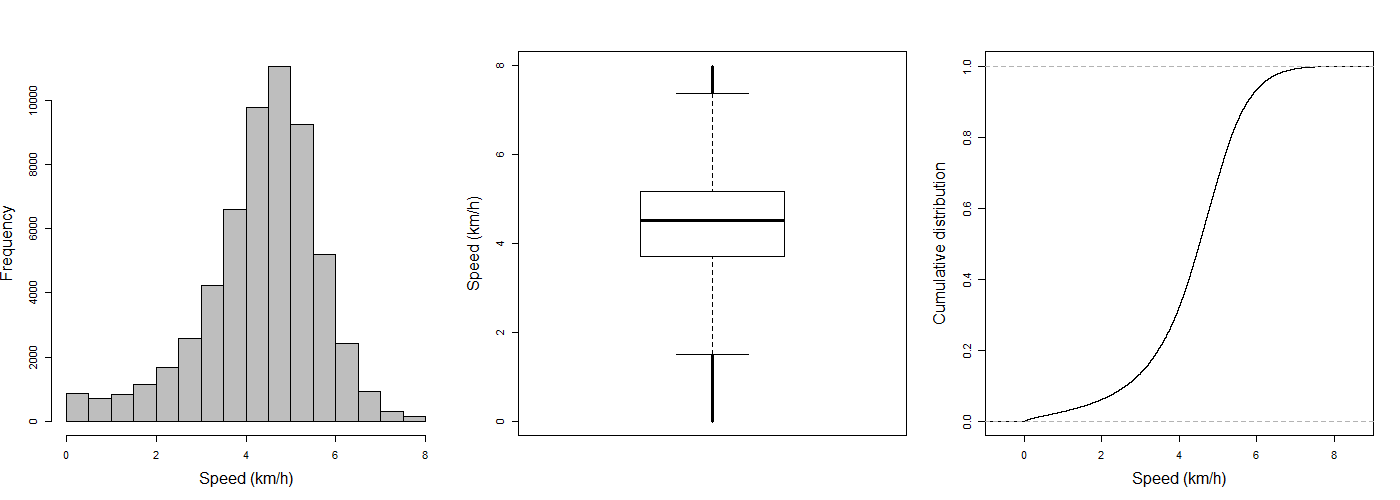 | | |
| **Slope** | 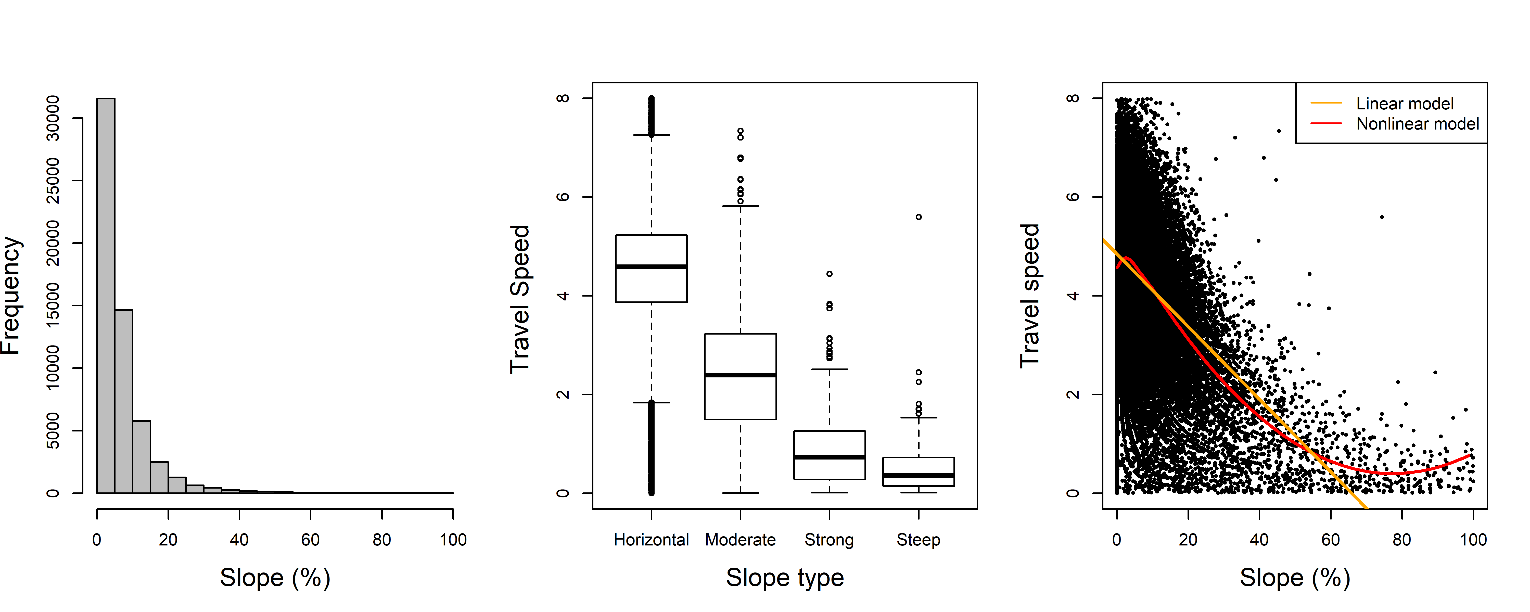 | | |
| **Rainfall** | 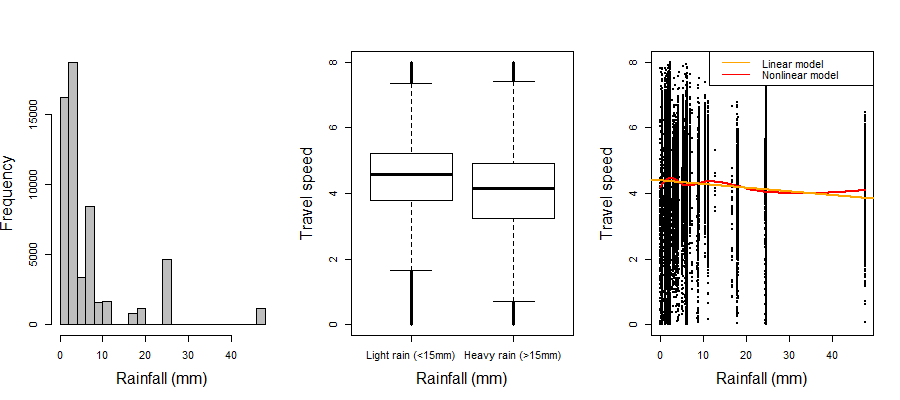 | | |
| **Distance** | 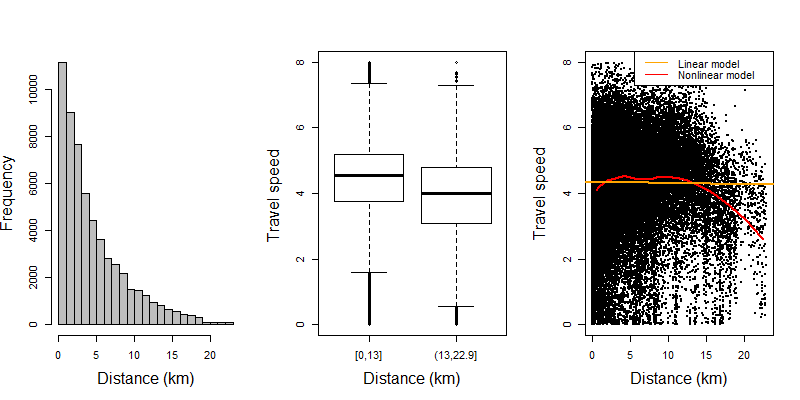 | | |
| **B** | | | |
| **Land cover** | 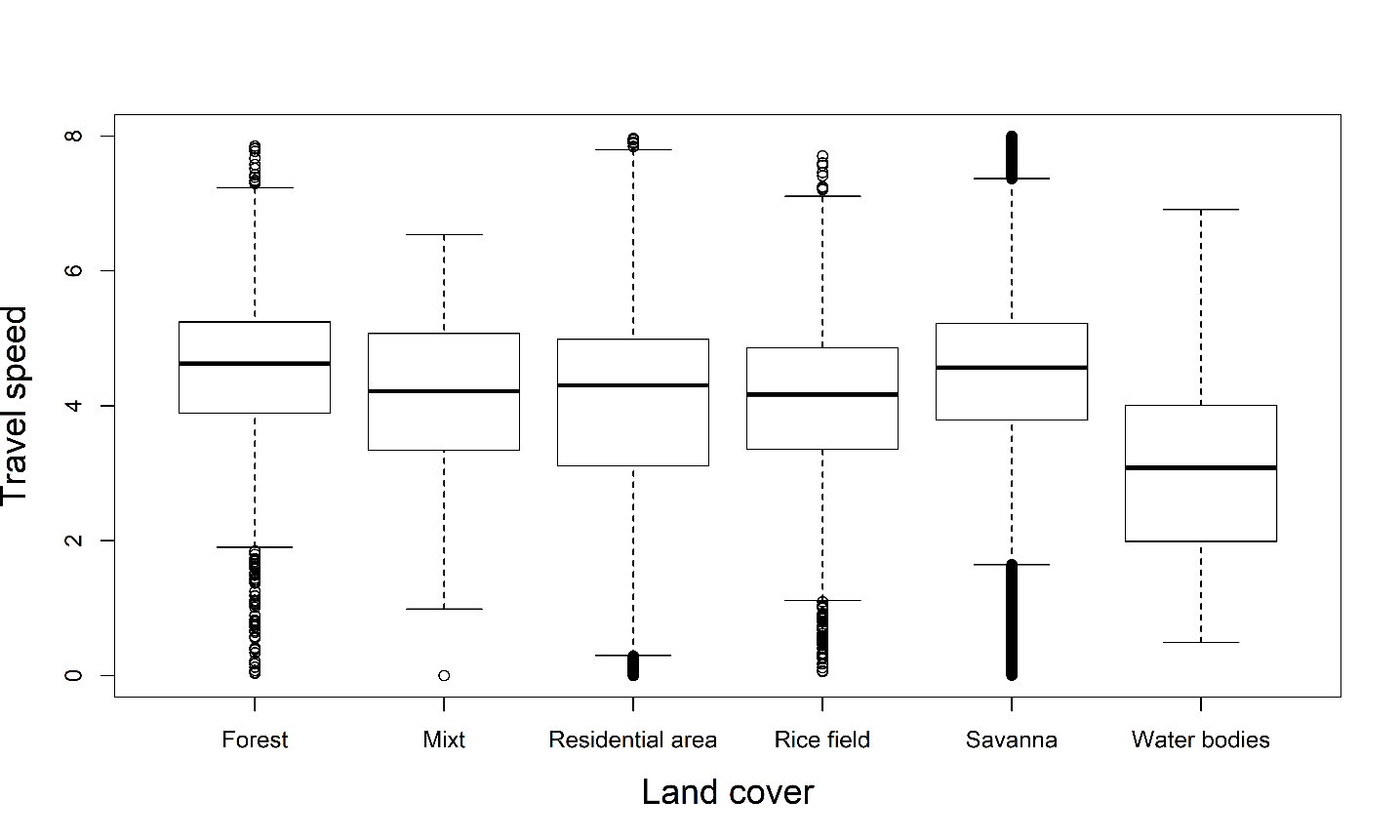 | | |
| **Individual type** | 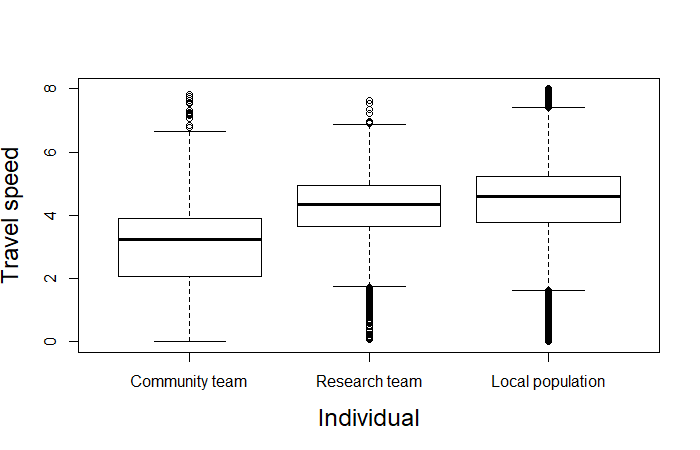 | | |
