# Supplementary material for: Improving geographical accessibility modeling for operational use by local health actors
Source: Int J Health Geogr. 2020 Jul 6;19:27. doi: 10.1186/s12942-020-00220-6 (PMC7339519; doi:10.1186/s12942-020-00220-6)

Additional file 3: Map of land cover in Ifanadiana district, 2018. Map was produced by a combination of remote sensing analysis and OSM mapping, providing 5 classes with a resolution of a least 10m (OSM classes were more precise, with a resolution <10m).

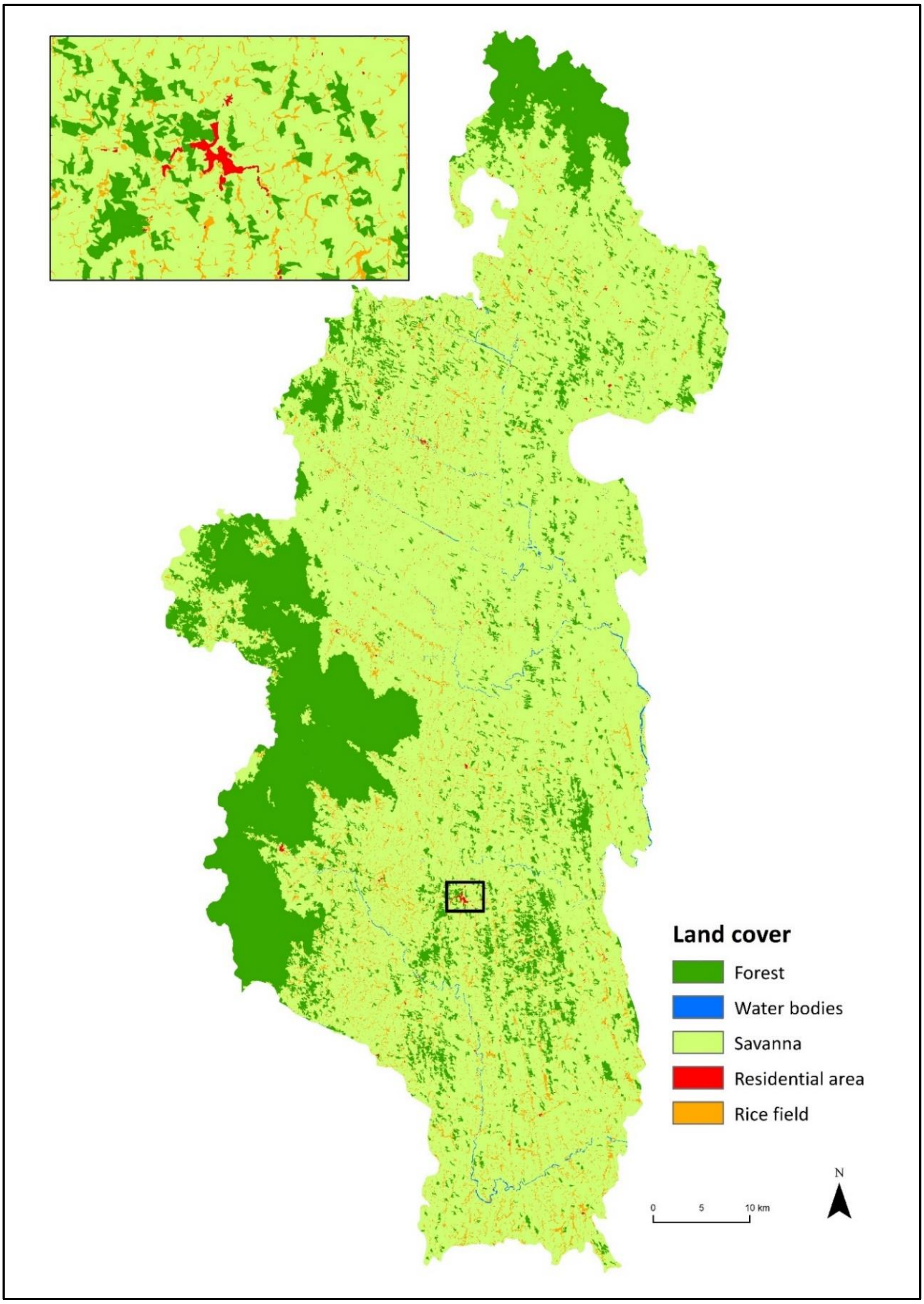

Supplement: Supplementary file 3 — Additional file 3. Map of land cover in Ifanadiana district, 2018, at a 10 m resolution following remote sensing analyses. [file 12942_2020_220_MOESM3_ESM.pdf]
