# Supplementary material for: Improving geographical accessibility modeling for operational use by local health actors
Source: Int J Health Geogr. 2020 Jul 6;19:27. doi: 10.1186/s12942-020-00220-6 (PMC7339519; doi:10.1186/s12942-020-00220-6)

Additional file 4: Histogram of travel distance and time to PHC and CHS. Pink histogram represents the number of populations to join CHS and green histogram the number of populations to join PHC. A) Shown the population per each class of distance. B) represents the number of populations per each class of travel time.

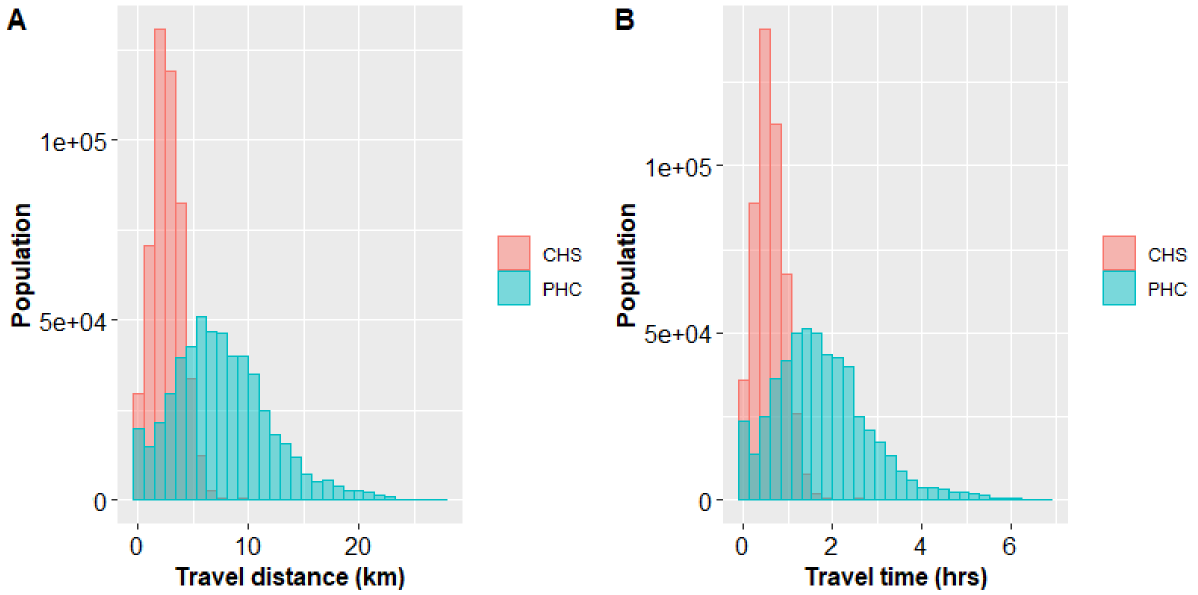

Supplement: Supplementary file 4 — Additional file 4. Histogram of travel distance and time to PHC and CHS. [file 12942_2020_220_MOESM4_ESM.pdf]
