# Supplementary material for: Improving geographical accessibility modeling for operational use by local health actors
Source: Int J Health Geogr. 2020 Jul 6;19:27. doi: 10.1186/s12942-020-00220-6 (PMC7339519; doi:10.1186/s12942-020-00220-6)

Additional file 5: Cumulative percentage of the population at each distance and time to seek treatment from the closest primary healthcare center (PHC, orange) and community health site (CHS, green).

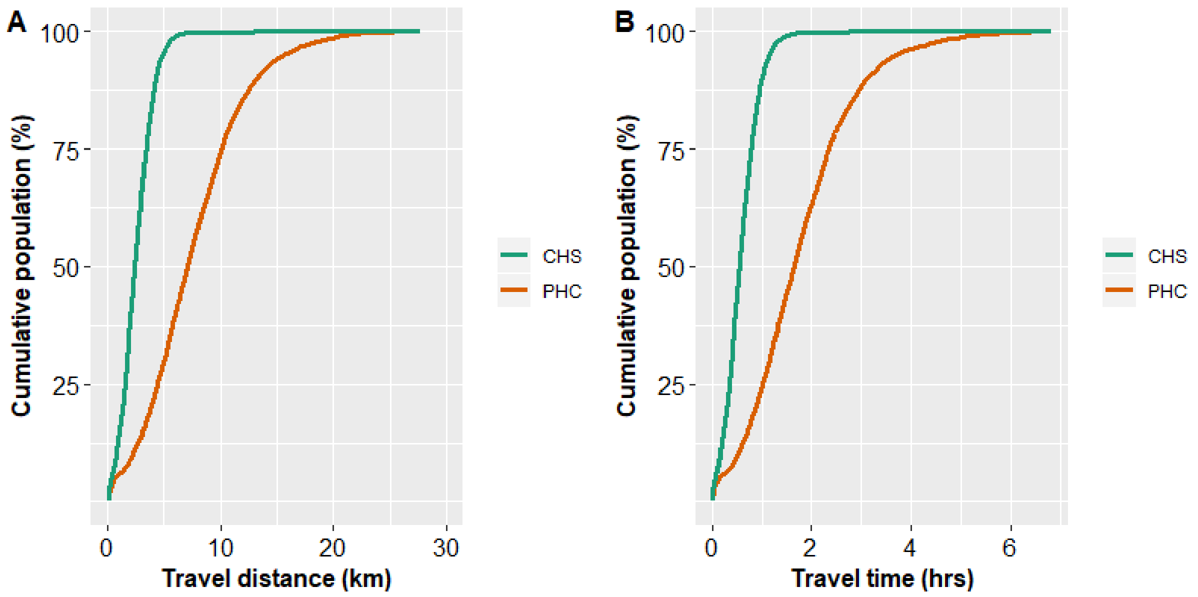

Supplement: Supplementary file 5 — Additional file 5. Cumulative percentage of the population at each distance and time to seek treatment from the closest PHC and CHS. [file 12942_2020_220_MOESM5_ESM.pdf]
