# Supplementary material for: Improving geographical accessibility modeling for operational use by local health actors
Source: Int J Health Geogr. 2020 Jul 6;19:27. doi: 10.1186/s12942-020-00220-6 (PMC7339519; doi:10.1186/s12942-020-00220-6)
Supplement: Supplementary file 6 — Additional file 6. Non-linear smooth and 95% confidence intervals of the effect of slope change (%, absolute values) on travel speed in the multivariate GAM model presented in Table 3. [file 12942_2020_220_MOESM6_ESM.docx]

**Additional file 6:** Non-linear smooth and 95% confidence intervals of the effect of slope change (%, absolute values) on travel speed in the multivariate GAM model presented in Table 3 on the main text.

**
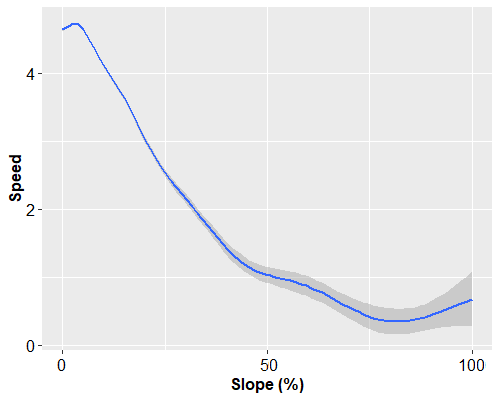
**
