# Supplementary material for: Improving geographical accessibility modeling for operational use by local health actors
Source: Int J Health Geogr. 2020 Jul 6;19:27. doi: 10.1186/s12942-020-00220-6 (PMC7339519; doi:10.1186/s12942-020-00220-6)

**Additional file 7**: Comparison of travel time with other commonly used methods. A) represent the relative difference with time estimated in the 168 fieldwork routes. B) and C) represent the difference with the 41,000 routes to PHC and CHS, using our estimates (main text) as reference.


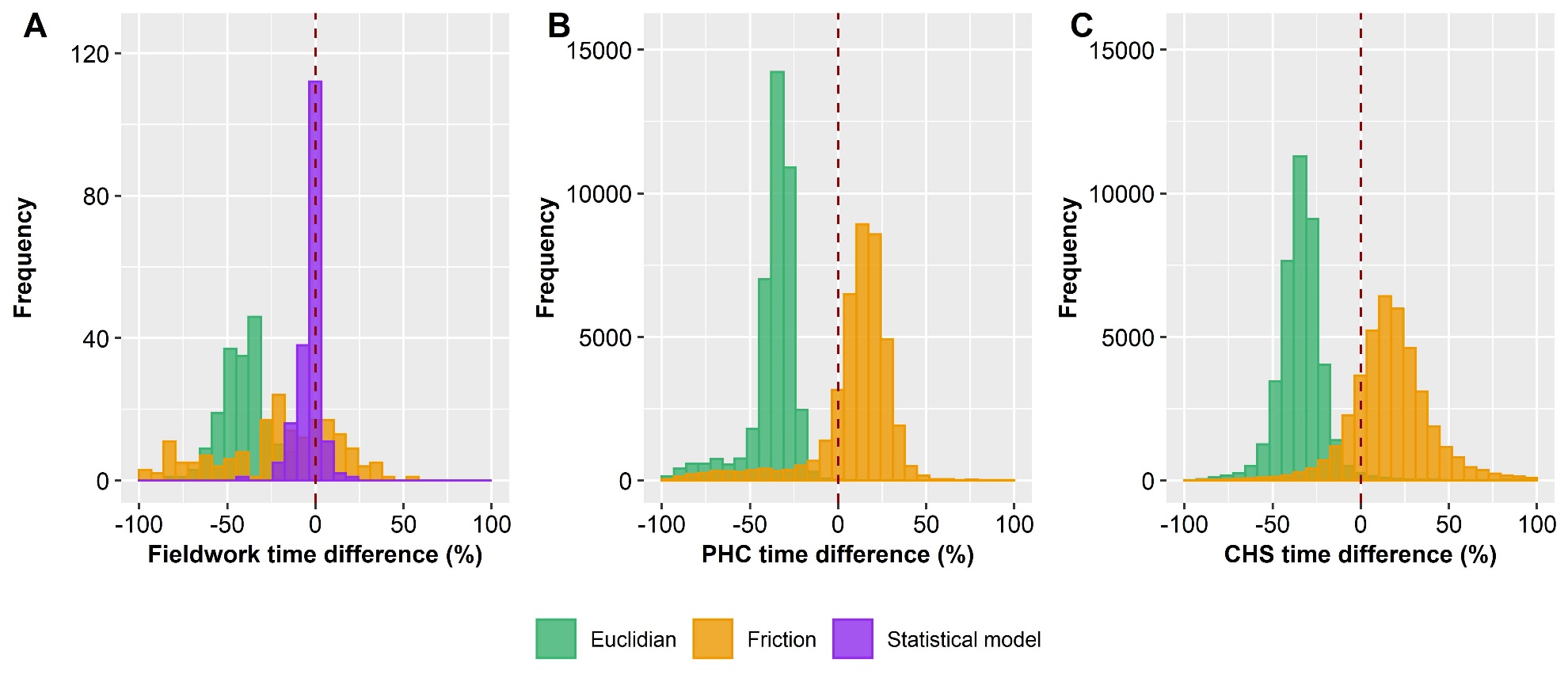

Supplement: Supplementary file 7 — Additional file 7. Comparison of travel time with other commonly used methods in geographic access modeling. [file 12942_2020_220_MOESM7_ESM.docx]
