# Supplementary material for: Improving geographical accessibility modeling for operational use by local health actors
Source: Int J Health Geogr. 2020 Jul 6;19:27. doi: 10.1186/s12942-020-00220-6 (PMC7339519; doi:10.1186/s12942-020-00220-6)
Supplement: Supplementary file 8 — Additional file 8. Comparison of travel time for the population of Ifanadiana between a scenario with 19 PHCs and a scenario with 21 PHCs, after the construction of two new PHCs in 2016 and 2018. [file 12942_2020_220_MOESM8_ESM.docx]

**Additional file 8**: Comparison of travel time for the population of Ifanadiana between a scenario with 19 primary healthcare centers (PHCs) and a scenario with 21 PHCs, after the construction of two new PHCs in 2016 and 2018.

**
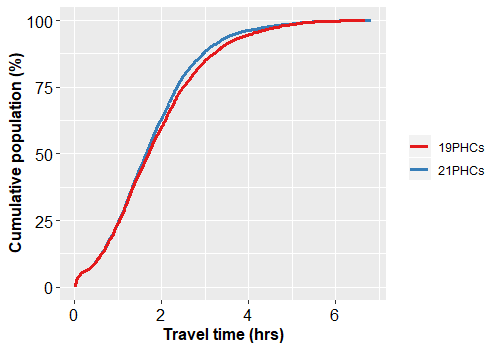
**
