# Supplementary material for: Improving geographical accessibility modeling for operational use by local health actors
Source: Int J Health Geogr. 2020 Jul 6;19:27. doi: 10.1186/s12942-020-00220-6 (PMC7339519; doi:10.1186/s12942-020-00220-6)

**Additional file 9**: Histogram of relative difference in travel time estimations for PHC and CHS routes between a statistical model with land cover (reference; as presented in the main text) and without land cover. Estimations of travel time without land cover were very similar to those with land cover, but in rare cases it led to differences between -50 and +25%.


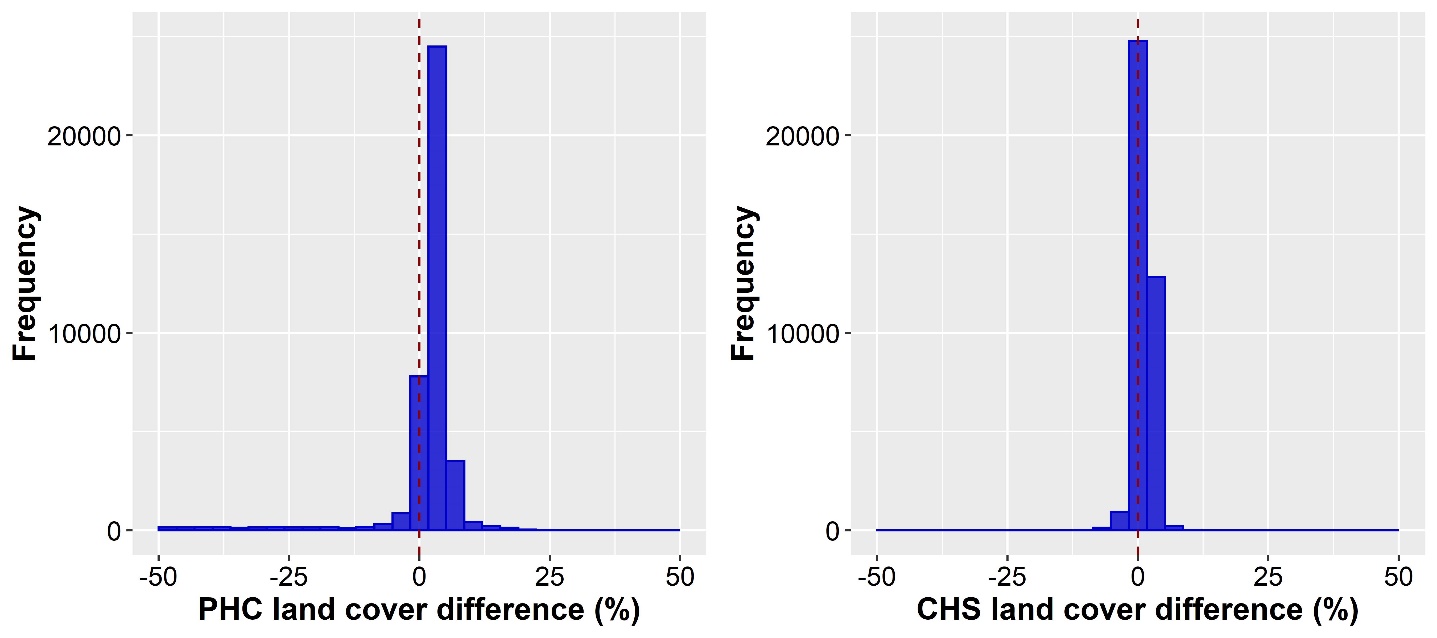

Supplement: Supplementary file 9 — Additional file 9. Histogram of relative difference in travel time estimations for PHC and CHS routes between a statistical model with land cover and without land cover. [file 12942_2020_220_MOESM9_ESM.docx]
